# Complete Genome Sequence of Pandoraea pnomenusa TF-18, a Multidrug-Resistant Organism Isolated from the Rhizosphere of Rice (Oryza sativa L. subsp. *japonica*)

**DOI:** 10.1128/MRA.01008-19

**Published:** 2020-01-02

**Authors:** Mihnea R. Mangalea, Emily K. Luna, Janet Ziegle, Christine Chang, Angela M. Bosco-Lauth, Richard A. Bowen, Jan E. Leach, Bradley R. Borlee

**Affiliations:** a Department of Microbiology, Immunology, and Pathology, Colorado State University, Fort Collins, Colorado, USA; b Department of Bioagricultural Sciences and Pest Management, Colorado State University, Fort Collins, Colorado, USA; c Pacific Biosciences, Menlo Park, California, USA; d Department of Biomedical Sciences, Colorado State University, Fort Collins, Colorado, USA; University of Maryland School of Medicine

## Abstract

Pandoraea pnomenusa strain TF-18 was isolated from the roots of rice seedlings on selective medium containing four classes of antibiotics for isolation of Burkholderia pseudomallei. Using Pacific Biosciences (PacBio) single-molecule real-time (SMRT) sequencing technology, we report here a complete genome of 5,499,432 bases, a GC content of 64.8%, and 4,849 coding sequences.

## ANNOUNCEMENT

The genus Pandoraea, referencing the Greek myth of Pandora’s box and the origin of sickness and misery, was described to classify ambiguous bacterial isolates cultured from cystic fibrosis patients (1). Relatively little is known about environmental Pandoraea pnomenusa strains; however, isolates have been reported to be capable of quorum sensing (2), oxalate degradation (3), and exopolysaccharide production (4). P. pnomenusa isolates were routinely found in association with rice plants during isolation from roots originating from independent seed lots and were preliminarily identified with published *P. pnomenusa*-specific primers (5).

*P. pnomenusa* isolates were cultured from the roots of the rice cultivar Kitaake (Oryza sativa L. subsp. *japonica*) via sonication (6) and grown on NAP-A selective medium (modified Ashdown’s medium containing gentamicin [4 μg/ml], norfloxacin [4 μg/ml], ampicillin [10 μg/ml], and polymyxin B [300 units/ml]) (7). Identification of the bacterial isolates as *P. pnomenusa* or a *Pandoraea* sp. was confirmed with matrix-assisted laser desorption ionization–time of flight mass spectrometry (MALDI-TOF MS) as previously described (8).

Genomic DNA was extracted from a representative isolate, *P. pnomenusa* TF-18. Individual colonies were grown on LB agar with no antibiotics at 28°C for 2 days and suspended in phosphate-buffered saline (PBS). The suspension was incubated at 37°C with 1% SDS and proteinase K (Sigma-Aldrich); 0.5 M NaCl was added, and cells were incubated at 65°C to ensure cell lysis. Genomic DNA was purified using phenol-chloroform-isoamyl alcohol (25:24:1), followed by washes with 95% ethanol and 80% ethanol. The pellet was resuspended in 10 mM Tris-HCl containing RNase A (0.1 mg/ml). Whole-genome sequencing was achieved in a 6-plex library using a long-read Pacific Biosciences (PacBio) single-molecule real-time (SMRT) sequencing platform. Libraries were prepared according to the published PacBio protocol (https://www.pacb.com/wp-content/uploads/Procedure-Checklist-Preparing-Multiplexed-Microbial-SMRTbell-Libraries-for-the-PacBio-Sequel-System.pdf) and sequenced on the Sequel system with v6.0 Sequel chemistry. The total number of reads was 30,617, and the mean read length was 36,707 bp. Assembly of the genome was conducted using the Hierarchical Genome Assembly Process (HGAP) v4 (9). Input reads were filtered to a minimum subread length of 500 bp. Final total coverage, including all subreads for the assembly, was 187× (mean) as determined by Arrow (PacBio, Menlo Park, CA). Quality assessment of the genome assembly was performed in QUAST (10) v5.0.2, and circularity of the final contig was determined by Circulator (11) v1.5.6.

The *de novo* sequenced genome of *P. pnomenusa* TF-18 was assembled into one contig with a GC content of 64.8%. Primary annotation of protein-coding sequences was performed using the NCBI Prokaryotic Genome Annotation Pipeline (PGAP) v4.8 (12), resulting in 4,849 coding regions, which included 99 pseudogenes, 4 copies of 5S rRNA, 4 copies of 16S rRNA, 4 copies of 23S rRNA, 67 tRNAs, and 4 noncoding RNAs (ncRNAs). Rapid Annotations using Subsystems Technology (RAST) v2.0 analysis (13) revealed 41 genetic features associated with “resistance to antibiotics and toxic compounds,” including five multidrug resistance efflux pumps and one predicted beta-lactamase. Analysis for secondary metabolites using antiSMASH (14) v5.0 revealed five biosynthetic gene clusters, which included two terpenes (a phosphonate and a phytoene), a bacteriocin, and two homoserine lactone synthases. Default parameters were used for all software unless otherwise specified. The genome presented here is valuable for understanding the molecular evolution of *Pandoraea* spp., as well as for bridging the gap between environmental and clinical isolates.

### Data availability.

The complete genome sequence of the bacterium Pandoraea pnomenusa TF-18 has been deposited in NCBI GenBank under accession no. CP042219. The NCBI BioProject is listed under accession no. PRJNA556667, and the raw sequence reads are listed under accession no. SAMN12362283. The strain is available from the corresponding author upon request.
